# The discovery that the human silencing hub is an epigenetic gatekeeper of innate immune regulation

**DOI:** 10.1186/s13100-026-00420-0

**Published:** 2026-11-04

**Authors:** Helen M. Rowe, Hale Tunbak, Rocio Enriquez-Gasca

**Affiliations:** 1 Centre for Immunobiology and Infection, Blizard Institute, Queen Mary University of London, London, E1 2AT UK; 2 Present Address: UCL Cancer Institute, University College London, London, WC1E 6BT UK; 3 Present Address: Barts Cancer Institute, Queen Mary University of London, London, EC1M 6BQ UK; 4 Present Address: The Francis Crick Institute, 1 Midland Road, London, NW1 1AT UK

This discovery story began, like most projects, in Didier Trono’s lab. As postdocs, Didier gave us a lot of freedom and he supported us in having side projects, and I had several of those, one of which was close to my heart. The goal was simple: To determine if the epigenetic pathways that we had discovered to silence transposable elements such as LINE-1 elements, in early development were operating in adult tissues. The quest was substantive: to identify regulators of autoimmunity that could be exploited to counteract immune-driven pathologies. The pathway that we were focusing on at that time was TRIM28, which we had uncovered to partner with sequence-specific KRAB-zinc finger proteins (ZFPs) to safeguard the genome from endogenous retroviruses and LINE-1 elements [1]. That original discovery was an extension of work that Daniel Wolf had undertaken in Steve Goff’s lab, where he pinpointed the TRIM28/KRAB-ZFP pathway to restrict the replication of the retrovirus, murine leukaemia virus [2].

To address the question of epigenetic silencing in adult tissues, we opted to use human peripheral blood mononuclear cells (PBMCs) from healthy individuals, and pieces of DNA from endogenous retroviruses that were TRIM28-repressed in embryonic cells that we had cloned into lentiviral vectors as reporter sequences. We had a protocol in Didier’s lab to allow us to introduce lentiviral reporter constructs into PBMCs (through first activating and then resting the cells), and the aim was to determine if the TRIM28-sensitive reporters were silenced compared to controls. However, the constructs that we had generated were sensitive to TRIM28 repression through mouse ZFPs in mouse embryonic stem cells. We lacked the appropriate tools to explore epigenetic silencing in human cells. Fortuitously, along came Chris Tie (CHCT), my first PhD student who was ​interested in UCL due to the high concentration of prestigious HIV research, including in Greg Towers lab, and he opted to undertake a PhD in my lab on TRIM28 regulation of endogenous retroviruses and innate immunity. His PhD goals are nicely summarised in his review article published in *Current Opinion in Virology* [3]. Chris had all the qualities that a scientist needs: curiosity, perseverance, integrity and serenity! He had a challenging project because, as mentioned above, he needed to generate new tools from scratch to dissect epigenetic silencing of human endogenous retroviruses. He also setup assays to measure the collateral effects of de-repression of endogenous retroviruses on innate immune responses. The latter objective was helped by us obtaining interferon reporter human cell lines from Jan Rehwinkel and through the helpful expertise from Greg Towers’ (Chris’ second supervisor), whose lab were employing transient transfection of interferon reporter plasmids to study cGAS/STING activation by HIV.

Chris found in his manuscript (published in *EMBO Reports* [4]) that (1) Binding of TRIM28 and enrichment of H3K9me3-heterochromatin is conserved in human adult tissues; (2) TRIM28 is required for endogenous retroviral silencing in PBMCs and human cell lines because its depletion leads to de-repression of several endogenous retroviruses (HERV-K, HERV-T and HERV-S) and loss of histone and DNA methylation, and (3) Loss of TRIM28 leads to nucleic acid sensing that is dependent on the adaptor protein MAVS, an effector of RNA sensing and interferon signalling.

Towards the end of Chris’ PhD, he did a mini-screen to determine which epigenetic repressor might play a dominant role as an epigenetic gatekeeper of type I interferon (IFN-I) regulation. He discovered that MPHOSPH8 (MPP8), a chromodomain component of the human silencing hub (HUSH) is responsible for negative regulation of IFN-I (published in Chris’ thesis [5]). This was very exciting news for us at the time, particularly because we were running a parallel project aimed at exploring whether HUSH plays a role in silencing endogenous retroviruses in early development. We had just pinpointed it to be required specifically for silencing of intact evolutionarily young LINE-1 elements in mouse and human embryonic cells. Suddenly, the stars were aligning with the observations in our two projects. Young (more recently integrated) LINE-1 elements were those most likely to generate nucleic acids as a stepping stone in their replication cycle that might induce viral mimicry. This latter project was led by Luisa Robbez-Masson (LRM), a postdoc in my lab, although since it was a big project, we all worked on it full-time through a Wellcome Trust Fellowship award that I had obtained to setup my lab. This included help from Connor Husovsky (CH) as a research assistant at that time who focused on the DNA methylation assays. We were happy to have that work accepted in *Genome Research* [6]. That project involved an important collaboration with Paul Lehner, whose lab (led by Iva Tchasovnikarova and Richard Timms) had discovered the HUSH complex in 2015 and had found it to mediate position-effect variegation in human cells and to contribute to HIV latency [7]. Of note, our own projects would not have been possible without support with the computational analyses from Javier Herrero and Lucia Conde at UCL and further help from all the co-authors.

Simultaneously, Joanna Wysocka’s lab and Yoshi Shinkai’s lab published manuscripts detailing that the HUSH complex safeguards against LINE-1 elements [8, 9], which in their case, they had discovered through genome-wide CRISPR screens. As a Principal Investigator who had recently setup my lab with a Wellcome Trust Fellowship and who had a small team, it was hugely inspiring for my team members and I to be publishing papers that were tackling similar questions to trailblazer PIs in the epigenetics field, such as Joanna Wysocka.

After Chris’ PhD, he moved on to working in the financial sector and I was left holding the ‘HUSH regulates IFN’ project. I didn’t mind temporarily though: since I love discovery science and jumping genes, figuring out the biology of sterile interferon responses that hit the roof was fun. I was relieved, however, when I was awarded a small Rosetrees grant focused on that project, and I also obtained an ERC starting grant. Through these awards, I could recruit a talented lab-based PhD student, Hale Tunbak (HT) and a talented computational postdoc, Rocio Enriquez-Gasca (REG) who were both dedicated to working full-time on the HUSH-IFN project. Hale made some important findings at the time, including showing that type I interferon responses induced following HUSH-depletion were mainly dependent on the RNA sensor MDA5, rather than on the DNA sensor, cGAS in several human cell lines, (although cGAS responses were also detected). We accumulated the bulk of the data for the manuscript, while my lab was still based at UCL. Poppy Gould (PG), James Holt (JH) and Liane Fernandes (LF), who were members of my lab at the time also helped with the manuscript and contributed to the scientific discussions. At UCL, we got valuable help with a few experiments from Petra Mlcochova in Ravi Gupta’s lab, who was working on DNA damage and from my husband, Pierre Maillard, who was working on anti-viral RNA interference. Specific reagents/assays were only possible thanks to external collaborations with some of the co-authors.

Once I moved the lab to QMUL in January 2020, we were rushing through the revisions of the manuscript and this overlapped with lockdown. Hale succeeded to accumulate evidence that overexpression of an engineered LINE-1 construct was sufficient to evoke an interferon response before we were locked out of labs. We were helped by the supportive work environment at the Blizard Institute and benefited from fruitful discussions with Immunologists, including David Leslie who was Hale’s second supervisor at QMUL for the remainder of her PhD. During the lockdown phase, Rocio maintained a relentless passion for the project and we were in constant dialog about it. In early lockdown, Rocio and I both enjoyed our discussions about outstanding questions in the manuscript, despite working being tricky at that time, especially for me with young kids. Rocio’s dedication never waned though, and she even established a number of new computational pipelines during that period. This included one to look at convergent transcription that pinpointed which L1HS element loci were most adept at generating overlapping sense/antisense RNAs, which might associate to form dsRNAs.

Our work was published in *Nature Communications* detailing HUSH as a gatekeeper of type I interferon regulation [10]. The main highlights of our study are summarised here: If you disrupt the HUSH complex in human cell lines and primary cells (primary fibroblasts) through depletion of MPP8 or Periphilin, there is a potent type I interferon response that is dependent on RNA sensing, secretion of interferon-beta, on the type I IFN receptor and on the JAK/STAT signalling cascade. Other pathways activated indirectly include DNA damage and the NFkB pathway, while zinc finger protein genes represent direct HUSH targets, supporting prior findings from Paul Lehner’s lab. Loss of HUSH leads to an increase in LINE-1 RNA and proteins as well as double-stranded RNAs (dsRNAs) accumulating in the cytoplasm as detected by dsRNA-specific antibodies (K1 and J2 antibodies). Endogenous retroviruses are derepressed too including ERV9 (LTR12), HERVH and HERVK, which are situated genomically significantly closer to interferon response genes than other genes. This latter observation suggests that the HUSH complex could regulate both the induction of interferon responses and the magnitude of the resulting IFN response; the first through nucleic acid sensing and the second due to loss of MPP8 unveiling a subset of LTRs that might act as enhancers for IFN response genes. Regarding LINE-1 elements, it is mainly young primate-specific LINE-1s (including L1HS) that are de-repressed. The IFN-I response is in part controlled through LINE-1 elements because dampening global LINE-1 expression inhibits IFN responses to HUSH-depletion, while overexpression of an engineered LINE-1 construct is sufficient to induce an IFN response. However, since loss of HUSH is accompanied by a plethora of retroelements being activated, including Alus, SVAs and ERVs, these latter retroelements could all contribute to nucleic acid sensing. Finally, loss of the HUSH component, MPP8 occurs in diverse cancers, where it correlates with in vivo IFN responses in cancer patients. It is notable that although HUSH repression links to other co-operating silencing pathways such as TRIM28 [6] and the nuclear exosome targeting (NEXT) RNA decay pathway [11, 12], removal of the HUSH subunit, MPP8 alone is sufficient to de-repress LINE-1 elements, correlating with IFN responses even in primary fibroblasts [10].

We have been working on and following the HUSH literature ever since (reviewed in [13, 14]. This includes the notable discovery of the HUSH-related complex, HUSH2 by Iva Tchasovnikarova’s lab and Paul Lehner’s lab, and Peter Lewis’ lab [15, 16], which from our own studies, we think is important in early development [17]. The only component unique to HUSH2 is TASOR2, which may be critical in dictating its unique binding profile to interferon-stimulated gene promoters and to KRAB-ZNF genes. HUSH, in contrast, targets mainly LINE-1s and other retroelements, indicating that TASOR may be critical in determining HUSH localisation. MPP8 and PPHLN1 are core HUSH components common to both HUSH and HUSH2 complexes [13]. These latter discoveries solve the puzzle of why depleting MPP8 or PPHLN1 but not TASOR leads to a potent type I interferon response in human cell lines: Depleting TASOR would promote more HUSH2 formation and suppression of interferon-response genes, whereas loss of MPP8 or PPHLN1 leads to de-repression of LINE-1s and nucleic acid sensing and leaves interferon-response genes poised for their activation through RNA and DNA sensing pathways.

Several other advances have been pioneered regarding the mechanisms of the HUSH complex targeting to chromatin and its biochemistry [18–21]. We and other labs are working on dissecting the retroelement-derived nucleic acid mediators of type I interferon induction, which is an area of research with potential broad relevance to cancer and autoinflammatory diseases. Of current interest in the field is the ability of LINE-1 elements to generate immunostimulatory RNA: DNA hybrids through ORF2 activity, which can engage the cGAS/STING pathway to activate IFN-I [22]. My lab has focused mainly on dissecting how retroelement-derived RNAs at the locus-specific level contribute to MDA5-driven autoinflammation, which is at the heart of tissue damage and autoimmune conditions.

Our own projects on HUSH and IFN responses are only the tip of the iceberg though, and there is still so much waiting to be discovered regarding the biochemistry of the HUSH complex, innate immunity and LINE-1 elements. Since LINE-1 elements are associated with autoimmune diseases and cancer [23], the ultimate question is how all this emerging knowledge could be utilized to pioneer new immunotherapies. There is a lot of interest in inducing viral mimicry in tumours using epigenetic inhibitors, for example, in combination with immune checkpoint blockade to enhance anti-tumour immune responses: For example, immune responses to glioblastoma were recently enhanced by targeting an epigenetic regulator, ZFP638 [24]. Understanding and targeting epigenetic checkpoints is a long-term area of research that my lab hopes to contribute to in our new base at the UCL Cancer Institute.

## Supplementary Information

Below is the link to the electronic supplementary material.

Supplementary Material 1

## Data Availability

No datasets were generated or analysed during the current study.
